# Editorial: Mitophagy and mitochondrial proteostasis in cardiovascular diseases

**DOI:** 10.3389/fcvm.2022.1024917

**Published:** 2022-09-23

**Authors:** Dorota Dymkowska, Valentina Parra

**Affiliations:** ^1^The Laboratory of Cellular Metabolism, Nencki Institute of Experimental Biology (PAS), Warszawa, Poland; ^2^Advanced Center of Chronic Diseases (ACCDiS), Facultad de Ciencias Químicas y Farmacéuticas y Facultad de Medicina, Universidad de Chile, Santiago, Chile; ^3^Departamento de Bioquímica y Biología Molecular, Facultad de Ciencias Químicas y Farmacéuticas, Universidad de Chile, Santiago, Chile; ^4^Red para el Estudio de Enfermedades Cardiopulmonares de Alta Letalidad (REECPAL), Universidad de Chile, Santiago, Chile

**Keywords:** cardiovascular disease, mitophagy, inflammation, energy metabolism, degradation mechanisms

Cardiovascular diseases (CVD) develop as the effect of heart or blood vessel failures. According to the World Health Organization (WHO) an estimated 17.9 million people died from CVD in 2019, representing 31% of all global deaths, 85% of which are due to heart attacks and stroke (1). It is widely recognized that mitochondrial malfunction is an early and prominent sign of myocardial damage. Mitophagy is an evolutionarily conserved cellular process that involves engulfing impaired or superfluous mitochondria, which are then degraded by lysosomes (2). Mitochondrial proteostasis is a dynamic balance in mitochondrial protein synthesis, transport, localization, expression, and degradation.

Dynamic regulation of metabolic phenotype also determines the maintenance of metabolic health and normal weight. Such homeostasis seems to be particular for alleviation of the risks of atrial fibrillation (Zhao et al.). However, mitochondria were also implicated in myocardial cell death caused by oxidative stress or calcium overload, both directly related to inflammation. The important role of mitochondria in the development of various failures is widely discussed. The dynamics of mitochondria linked with efficient mitophagy were shown to play a key role in cellular metabolism. The disturbances in mitochondrial metabolism and function were documented to be involved in the development and progression of cardiometabolic diseases (Lin et al.) including coronary heart disease and myocardial injury (Liu and Wu). Mitochondrial quality control mechanisms regulate the morphology and structure of mitochondria to ensure the energetic requirements of cardiomyocytes not only under physiological conditions but in response to stress. Mitochondria may act as regulatory factors and are involved in the inflammatory response of cells as they produce reactive oxygen species (ROS). ROS may play the role of signaling molecules that transmit stress signals initiated by the activation of TLR4, but may also be responsible for mitochondrial dysfunction development as they cause proteins, lipids and DNA damage or point mutation causing acute metabolic consequences (Li et al.).

In response to damage from transient hypoxia or mild oxidative stress, the mitochondrial protein quality control machinery is activated to maintain the diversity and function of mitochondrial proteins through the activity of chaperones and proteases, and induction of the mitochondrial unfolded protein response (3). When damaged mitochondria cannot be repaired, they are degraded through mitophagy in a receptor-dependent or independent manner. Therefore, it is acknowledged that efficient mitophagy and mitochondrial proteostasis are vital for mitochondrial function and concomitant myocardial performance. During CVD, such as myocardial infarction, sepsis-related myocardial depression, heart failure, and diabetic cardiomyopathy, mitochondrial proteostasis is disrupted. The effect is accompanied by the accumulation of unfolded or abnormal proteins within mitochondria, contributing to mitochondrial damage and subsequent cardiomyocyte dysfunction.

Elevated levels of glucose or fatty acids that are common risk factors in diabetes affect mitochondrial function and favor diabetic cardiomyopathy development. The deregulation of energy metabolism is also associated with the upregulation of CyP4501A1 targeted to mitochondria (Chen et al.). In turn, in septic cardiomyopathy, the mitophagy suppression was correlated with the elevated expression of Receptor-Interacting Protein Kinase 3 which also enhances response to LPS (Zhu et al.).

The relationship between effective mitophagy and mitochondrial function and ultrastructure certainly needs further exploration for a deep and comprehensive understanding of the health ability of mitophagy and dynamic mitochondrial movements. Moreover, the protective effects of various drugs and natural compounds are also taken into account. One of such promising molecules is curcumin, which was shown to improve cardiopulmonary resuscitation due to alteration of myocardial dysfunction induced by ischemia/reperfusion injury. Curcumin was capable to ameliorate mitochondrial architecture and energy metabolism as well as exhibiting antioxidative properties. Finally, the inhibition of the mitochondrial pathway of apoptosis was observed as a consequence of the downregulation of uncoupling proteins (Zhang et al.).

There is no doubt, that efficient degradation processes like mitophagy are important for the functioning of the cardiac system. However, the particular mechanism or factors engaged in the development and progression of CVDs were not described in detail to date. Even though further studies are desired and important, the data presented in the Research Topic “*Mitophagy and Mitochondrial Proteostasis in Cardiovascular Diseases*” by Frontiers in Cardiovascular Medicine help us to better understand this issue.

## Author contributions

All authors listed have made a substantial, direct, and intellectual contribution to the work and approved it for publication.

## Funding

This work was supported by the National Science Centre (grant number 2015/19/B/NZ3/02302 to DD). Grant awarded to Krzysztof Zabłocki (Principal Investigator). This work was also supported by grants from the Agencia Nacional de Investigación y Desarrollo (ANID), Chile: FONDECYT grants 1190743 (to VP), ANILLO ACT210004 (to VP), FONDAP 15130011 (to VP). U-Redes G_2018-35 (to VP) and CRP-ICGEB CHL18-04 (to VP).

## Conflict of interest

The authors declare that the research was conducted in the absence of any commercial or financial relationships that could be construed as a potential conflict of interest.

## Publisher's note

All claims expressed in this article are solely those of the authors and do not necessarily represent those of their affiliated organizations, or those of the publisher, the editors and the reviewers. Any product that may be evaluated in this article, or claim that may be made by its manufacturer, is not guaranteed or endorsed by the publisher.
